# Combinatorial Binding Leads to Diverse Regulatory Responses: Lmd Is a Tissue-Specific Modulator of Mef2 Activity

**DOI:** 10.1371/journal.pgen.1001014

**Published:** 2010-07-01

**Authors:** Paulo M. F. Cunha, Thomas Sandmann, E. Hilary Gustafson, Lucia Ciglar, Michael P. Eichenlaub, Eileen E. M. Furlong

**Affiliations:** European Molecular Biology Laboratory, Heidelberg, Germany; Friedrich Miescher Institute for Biomedical Research, Switzerland

## Abstract

Understanding how complex patterns of temporal and spatial expression are regulated is central to deciphering genetic programs that drive development. Gene expression is initiated through the action of transcription factors and their cofactors converging on enhancer elements leading to a defined activity. Specific constellations of combinatorial occupancy are therefore often conceptualized as rigid binding codes that give rise to a common output of spatio-temporal expression. Here, we assessed this assumption using the regulatory input of two essential transcription factors within the *Drosophila* myogenic network. Mutations in either *Myocyte enhancing factor 2* (*Mef2*) or the zinc-finger transcription factor *lame duck* (*lmd*) lead to very similar defects in myoblast fusion, yet the underlying molecular mechanism for this shared phenotype is not understood. Using a combination of ChIP-on-chip analysis and expression profiling of loss-of-function mutants, we obtained a global view of the regulatory input of both factors during development. The majority of Lmd-bound enhancers are co-bound by Mef2, representing a subset of Mef2's transcriptional input during these stages of development. Systematic analyses of the regulatory contribution of both factors demonstrate diverse regulatory roles, despite their co-occupancy of shared enhancer elements. These results indicate that Lmd is a tissue-specific modulator of Mef2 activity, acting as both a transcriptional activator and repressor, which has important implications for myogenesis. More generally, this study demonstrates considerable flexibility in the regulatory output of two factors, leading to additive, cooperative, and repressive modes of co-regulation.

## Author Summary

While genetic studies are essential to reveal the phenotypic relationships between genes, it is often very difficult to disentangle the molecular mechanism of two genes that phenocopy each other. In this study, we used global scale and single gene analysis to investigate the relationship between two transcription factors whose mutant embryos have a similar defect in myogenesis. In *Drosophila*, *Mef2* mutant embryos display a block in myoblast fusion, which is very similar to what is observed in mutant embryos for *lmd*, a zinc-finger transcription factor. To understand the underlying nature of these defects we used ChIP-on-chip analysis to obtain a global view of their co-regulated enhancers, and we used expression profiling of mutant embryos to reveal their downstream transcriptional response. The results indicate that Lmd acts as a tissue specific modulator of Mef2 activity. Using *in vivo* and *in vitro* reporter assays, we show that co-binding to the same enhancer element can lead to diverse regulatory responses. The presence of Lmd has an additive, cooperative, or repressive effect on Mef2 activity, demonstrating that it acts as a molecular switch for gene expression during muscle differentiation. More broadly, our results highlight the difficulty in translating information on combinatorial binding data into a functional regulatory response.

## Introduction

Development is driven by precise patterns of spatio-temporal gene expression, which are regulated through the action of transcription factors and cell signaling cascades converging on *cis*-regulatory modules (CRMs). CRMs are typically bound by multiple transcription factors, whose concentrations and interactions change dynamically over time. It is this combinatorial and dynamic property of CRM occupancy which makes regulatory output difficult, if not impossible, to predict based on information from a single transcription factor (TF) [1]. Understanding the regulation of complex developmental processes requires linking combinatorial binding at the molecular level to the regulation of these processes at the phenotypic level. We have assessed the contribution of two well-studied TFs, Mef2 (Myocyte Enhancing Factor 2) and Lmd (Lame duck) to the cellular process of myogenesis during *Drosophila* development. Although the phenotypic defects in myoblast fusion are almost identical in *Mef2* or *lmd* loss-of-function mutant embryos [2]–[4], the molecular relationship between these TFs activity is poorly understood.

Members of the Mef2 family of MADS-box proteins were first characterized in vertebrates as important regulators downstream of the MyoD family of transcription factors, and have since been identified as part of an evolutionarily ancient regulatory network driving myogenesis from flies to man [5]. In vertebrates, Mef2 transcription factors act as central regulators of cell proliferation, survival, apoptosis and differentiation in a range of cell types, including skeletal, cardiac and smooth muscle, brain, neural crest, lymphocytes and bone (reviewed in [5]). This diversity in Mef2 function is achieved through regulation by extracellular signals and cooperative activity with specific co-regulators. In skeletal muscle, for example, Mef2 acts together with bHLH transcription factors to regulate the expression program that drives myogenic differentiation [6], [7]. In neural crest cells, Mef2c acts cooperatively with the DLX5 and DLX6 homeodomain TFs to regulate craniofacial development [8], [9], while in smooth muscle cells Mef2 acts together with myocardin [10]. Thus, Mef2 TFs have little inherent instructive potential by themselves but rather act together with tissue-specific TFs to drive specific gene expression programs. Given the diverse roles of the Mef2 gene family during development, many more co-regulators are likely to be required to generate the spectrum of transcriptional responses elicited by these factors.

In *Drosophila* embryos, the single *Mef2* ortholog is expressed exclusively in mesoderm and its muscle derivatives. Even in this relatively simple model system, Mef2 regulates distinct batteries of target genes in precise spatial patterns (e.g. in the dorsal vessel, the somatic mesoderm and visceral mesoderm [11], [12]), and in a specific temporal order [11], [13]. Global *in vivo* occupancy experiments revealed dynamic Mef2 enhancer binding; although Mef2 is expressed continuously, it binds to one group of enhancers only early in development and to another group only at late developmental stages [11]. The temporal shift in the expression onset of Mef2 target genes [11], [13] as well as their spatial diversity, indicates a requirement for co-regulators, similar to the mechanism of Mef2 action in vertebrates [5]. *holes-in-muscle* (*him*) was recently identified as a potential repressor of Mef2-dependent transcriptional activation via the recruitment of the general co-repressor Groucho [14]. Regulation by Him therefore provides one mechanism to alter the temporal output of Mef2 activity once it is bound to an enhancer. However, other co-regulators are clearly required to modulate Mef2's temporal enhancer occupancy and to restrict its spatial activity.

The *Drosophila* body wall muscle or somatic muscle is formed from two heterogeneous populations of cells- the founder cells (FCs), which represent 30 distinct cells in each hemisegment of the embryo, and the fusion competent myoblasts (FCMs) [15]. Once specified, a single FC will fuse with a defined number of FCMs to give rise to a syncytial myotube of distinct identity, defined by its size, shape and attachments. Mef2 is required to initiate a program that regulates myoblast fusion and drives the differentiation program of the resulting myotube into a contractile myofiber [2]. The zinc-finger transcription factor Lame duck maintains *Mef2* expression in FCMs, and like *Mef2*, is also essential to regulate a program of muscle differentiation, the first step of which is myoblast fusion [3], [4], [16]. *lmd* mutant embryos have defects in the specification or maintenance of FCMs [4], which results in an expansion of Zfh1-expressing pericardial cells and adult muscle precursor-like cells [17]. In contrast to Mef2, the molecular function of Lmd is more poorly understood; its only known direct target gene being *Mef2* itself [3].

Given the extensive co-expression of *lmd* and *Mef2* and the similarity in the myoblast fusion phenotype observed in their loss-of-function mutants, we hypothesized that Lmd may act as an FCM-specific modulator of Mef2 activity. To assess this, we have systematically compared the *in vivo* enhancer occupancy of Lmd and Mef2 and identified a large number of combinatorially bound enhancers during myogenesis. Expression profiling of loss-of-function *lmd* and *Mef2* mutants revealed that, although these TFs co-occupy the same enhancer region, they have different regulatory effects on the expression of the target genes. We used a combination of *in vivo* and *in vitro* approaches to demonstrate differential integration of regulatory input from Lmd and Mef2 at individual CRMs. Taken together, these data emphasize the diversity of transcriptional responses that can be generated by two transcription factors and identify Lmd as a new context-specific modulator of Mef2 activity.

## Results

### Obtaining a systematic map of Lmd-bound enhancer regions *in vivo*

As a first step towards understanding the phenotype of *lmd* mutant embryos and its potential combinatorial regulation with Mef2, we set out to identify Lmd-bound enhancer regions and directly regulated target genes. To identify Lmd-bound enhancers within the developing embryo, we used chromatin immunoprecipitation followed by microarray analysis (ChIP-on-chip) during defined stages of muscle development. Lmd-associated DNA was precipitated from tightly staged embryos at two consecutive developmental time points, spanning most of the developmental stages when *lmd* is expressed (stages 10–13). To obtain data with high sensitivity and specificity we performed a total of eight independent chromatin immunoprecipitations per time-point using two different anti-Lmd antibodies for each time point (Materials and Methods). The enriched DNA sequences were analyzed on microarrays containing overlapping 3 kb fragments tiling across ∼50% of the *Drosophila* genome [11]. Genomic regions were considered bound by Lmd if they were significantly enriched with both antibodies, thereby reducing potential false targets caused by non-specific antibody effects.

**Figure 1:**
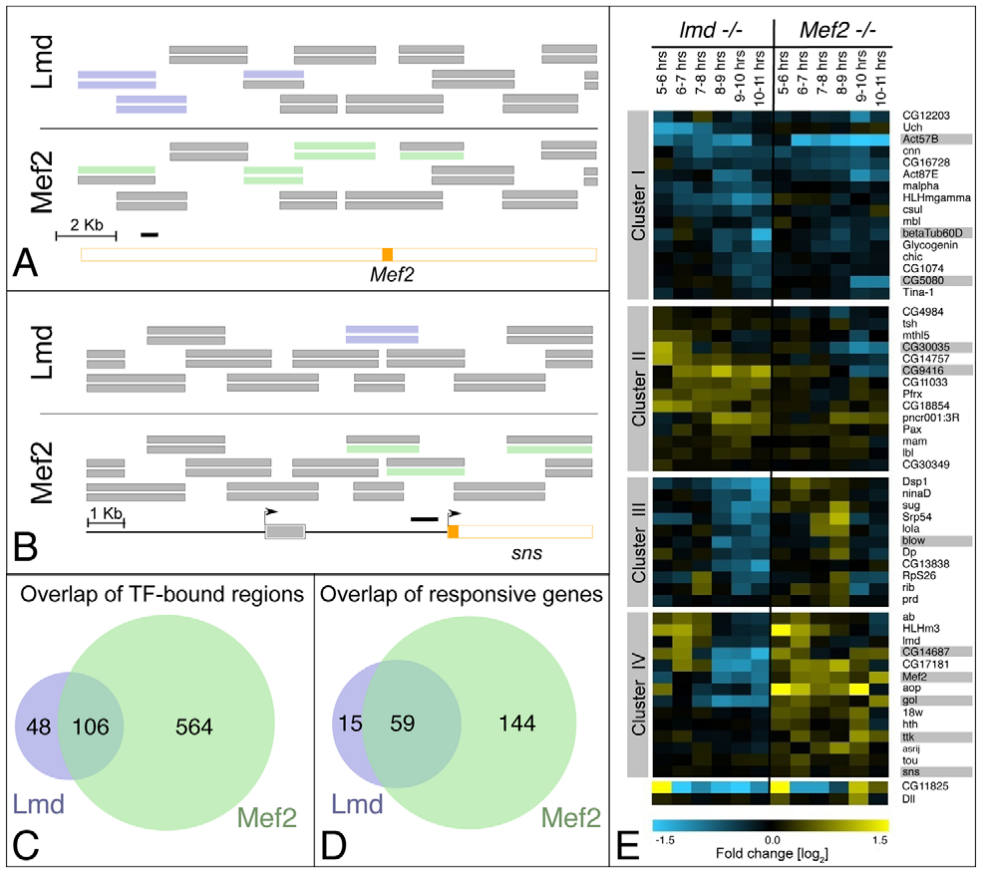
A global comparison of Lmd and Mef2 activity. (A) Schematic overview of a genomic region within an intron of the *Mef2* locus: An exon (located on the antisense strand) is shown in orange. Genomic fragments on the tiling arrays are indicated as stacks of two horizontal bars in their corresponding genomic position. The top bar represents the results from the 6–8 hour (stages 10–11) ChIP-on-chip time point, the lower one corresponds to the 8–10 hour (stages 12–13) time point. Significantly enriched regions are indicated in blue for Lmd (top) and in green for Mef2 (bottom). The black bar indicates the location of the previously identified Lmd-binding site. Both Lmd and Mef2 are co-bound to genomic regions overlapping the known Lmd binding site at the 6–8 hr time point, as well as to other sites in this area. (B) Schematic overview of the genomic region upstream of the *sns* locus (exons show in orange): The known sns enhancer (black bar) partially overlaps with tiling array probes bound by Lmd (at both time points, blue bars) and Mef2 (at 8–10 hour time point, green bar). Additionally, Mef2 binds to other locations upstream and intronic of the *sns* locus. (C) Lmd and Mef2 co-occupy many genomic locations: Venn diagram displaying the number of non-overlapping regions significantly enriched in Lmd ChIPs (blue) and significantly enriched in Mef2 ChIPs at the same stages of development (green). Both factors co-occupy a large number of regions (overlap). (D) Co-regulation of common direct target genes by Lmd and Mef2: The majority of Lmd target genes (blue) are co-regulated by Mef2 (overlap), while Mef2 regulates a large number of additional genes (green). (E) Differential gene expression in *lmd* and *Mef2* loss-of-function mutants [log_2_]: differences in expression between mutant and wt embryos were recorded in a timecourse for *lmd* (left) or *Mef2* (right) mutant embryos. Shown are all direct target genes of Lmd that are significantly misregulated at one or more time points in either mutant background (fold change >1.6, q<1%). K-means clustering was used to highlight similar downregulation (top) in both mutants, similar upregulation (centre) or divergent expression changes (bottom). Color scale indicates fold changes [log_2_]. Genes studied in more detail are marked in grey.

Lmd binding was detected at 154 unique genomic regions at one or both developmental time points (Table S1), including the only known Lmd-binding site upstream of the *Mef2* locus ([3]; Figure 1A). In addition, Lmd binds to a previously characterized enhancer of *sns* ([18]; Figure 1B), a transmembrane protein that requires *lmd* for its expression in FCMs [3] indicating that Lmd directly regulates *sns* expression. The expression of the bHLH transcription factor *twist* persists longer in *lmd* loss-of-function mutants than in wild-type embryos [4]. As the DNA-binding domain of Lmd is similar to the Gli-family of transcription factors, which can act both as transcriptional activators and repressors, it was proposed that Lmd may directly repress Twist [4]. However, no significant Lmd-binding was detected in the *twist* locus (data not shown). Although we cannot exclude low-level Lmd occupancy below the detection limit of our assay, this result suggests an indirect regulatory connection between Lmd and *twist*.

**Figure 2:**
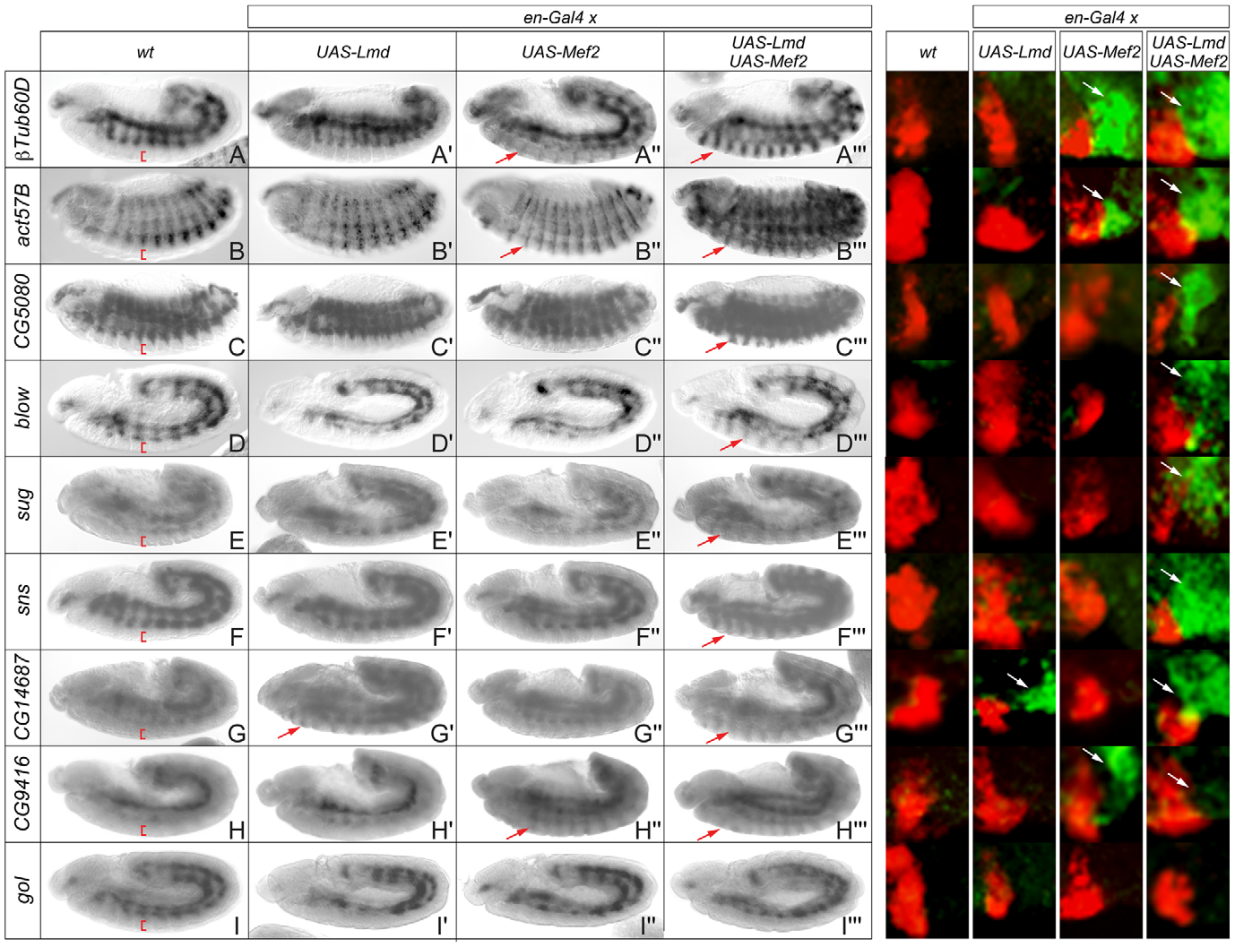
Ectopic expression of Lmd and Mef2 reveals differential regulatory influence on target gene expression. Left hand panels: Colorimetric *in situ* hybridization (black and white images). (A–I) *In situ* hybridization of wild-type embryos with probes specific for (A) *βTub60D*, (B) *act57B*, (C) *CG5080*, (D) *blow*, (E) *sug*, (F) *sns*, (G) *CG14687*, (H) *CG9416* and (I) *gol*, detecting specific expression in the mesoderm. No specific staining was observed in the ectoderm (red brackets). An engrailed-Gal4 driver line was used to ectopically express (A′–I′) UAS-Lmd, (A″–I″) UAS-Mef2-HA or (A′″–I′″) both UAS-Mef2-HA and UAS-Lmd in ectodermal stripes. Lmd and Mef2 show differential ability to activate ectopic target gene expression (red arrows). Expression of 8 of 9 genes can be induced in the ectoderm when both factors are present (A′″–H′″), while *gol* expression was never observed in the ectoderm (I′″). Right hand panels: Double fluorescent *in situ* hybridization of the same genotypes with probes specific for the corresponding genes (green) and *wingless* (red). A high magnification of the ectodermal *wingless* staining reveals adjacent ectopic staining in the *engrailed* domain (white arrows), not detected in wild-type embryos. Full embryos are shown in Figure S2.

The recovery of enhancers of both *Mef2* and *sns*, genes known to be genetically downstream of *lmd*, underscores the accuracy of the ChIP-on-chip results. Moreover, a number of Lmd-bound regions overlap previously characterized muscle enhancers, including *betaTub60D* [19], *Act57B* [20], *CG14687* and *CG9416* [11] (Figure S1 and Table S2) and are dependent on Lmd for their activity (see below). In addition, we have characterized the activity of four previously unknown Lmd-bound enhancers that are responsive to Lmd both *in vivo* (Figure 2) and *in vitro* (see below).

### Combinatorial binding of Lmd and Mef2 to shared *cis*-regulatory modules

**Figure 3:**
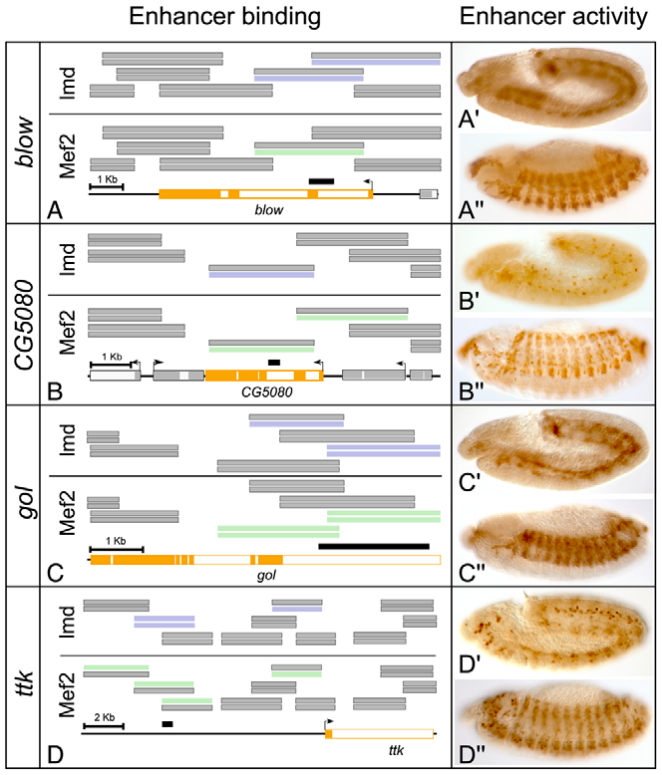
ChIP–bound regions function as enhancer elements capable of activating mesodermal expression *in vivo*. (A–D) Schematic overviews of the tiling array probes covering the (A) *blow*, (B) *CG5080*, (C) *gol* and (D) *ttk* loci. Regions significantly enriched in Lmd ChIPs (blue) and Mef2 (green) were selected and intergenic sequences (black bars) assayed for regulatory activity *in vivo*. (A′–D″) Immuno-histochemistry using an anti-GFP antibody to detect reporter gene expression. All four enriched sequences were able to specifically activate GFP expression in transgenic embryos in the mesoderm as early as (A′–D′) stage 11, with persistent GFP-presence at (A″–D″) stage 13.

The activities of Lmd and Mef2 are required for the initiation of myoblast fusion, presumably due to the regulation of a battery of genes essential for this process or for the identity of the FCMs themselves. To investigate potential co-regulation of target genes, we compared the enhancer binding data from Lmd (presented here) to our previously reported ChIP-on-chip data for Mef2 performed at the same developmental time points [11]. 106 out of 154 (68.8%) Lmd-bound regions are co-bound by Mef2 during muscle development (Figure 1C), suggesting that the majority of these regions are co-regulated by both TFs. This is likely to be a conservative estimate as regions bound by one or both transcription factors just below our thresholds are not considered. Nevertheless, the extensive level of enhancer co-occupancy (Figure 1C) indicates that combinatorial regulation by these two TFs is an important feature within the myogenic program. In many cases the temporal profile of Lmd and Mef2 binding to shared enhancers is identical, again indicating that these two TFs act together to co-regulate enhancer output. For example, both TFs only bind to the *ttk* (*tramtrack*) enhancer at stages 10–11, but not later, while the *blow* (*blown fuse*) and *CG5080* enhancers are co-bound at stages 12–13, and not earlier (Figure 3).

### Differential requirements of Lmd and Mef2 activity for target gene expression

The combinatorial binding of Lmd and Mef2 to shared CRMs raises several interesting questions. How much regulatory input does each factor contribute to the activation of an enhancer? Are Lmd and Mef2 acting in a co-operative or additive manner to regulate target gene expression? Are both transcription factors required for enhancer activation, or do they act redundantly? We have used several approaches, both *in vivo* and *in vitro*, to address these questions. First, we used global expression profiling to determine which genes require Lmd and Mef2 activity for their correct expression *in vivo* (Figure 1E).

We performed a developmental time-course of gene expression, comparing the transcriptional state of wild-type embryos to that of *lmd*-mutant embryos at six consecutive one-hour windows of development, providing a high-resolution map of *lmd*-dependent changes in gene expression. These experiments identified 640 genes that are genetically downstream of *lmd* during the stages of myoblast fusion and the initiation of terminal muscle differentiation (Table S3). By integrating this differential gene expression data with information on Lmd enhancer occupancy (ChIP-chip data) and muscle-specific gene expression patterns (from BDGP *in situ* hybridizations, [21]) we defined a high-confidence set of 74 target genes [11] that are likely to be directly regulated by Lmd (Table S1 and Table S4). Among these are a number of genes known to be involved in myoblast fusion, including *Mef2*, *sns* and *blow*, as well as genes with characterized roles in other aspects of muscle development, suggesting that Lmd may have a broader role in myogenesis than previously anticipated.

In a previous study, we used ChIP-on-chip experiments and expression profiling to identify a stringent set of Mef2 direct target genes at multiple stages of development [11]. Comparing these data to that of Lmd revealed that a large (79.7%) and highly significant (*p*<2.2×10^−16^, Fisher's exact test) proportion of Lmd direct target genes are also directly regulated by Mef2 (Figure 1D). Thus the majority of the regulatory input provided by Lmd is mediated in conjunction with Mef2, which is not the case in the other direction. Mef2 regulates many target genes independently of Lmd, reflecting its broader expression and role in muscle development (Table S4).

To assess the regulation of Lmd direct target genes we first examined their transcriptional response to loss of regulatory input from either Lmd or Mef2. 57 of the 74 Lmd direct target genes are differentially expressed in *lmd* and/or *Mef2* mutant embryos compared to stage-matched wild-type controls (visualized by K-means clustering in Figure 1E). This analysis revealed an unanticipated diversity in transcriptional responses, despite the fact that the majority of genes have an enhancer bound by both transcription factors. One group of genes (cluster I, Figure 1E) is downregulated in both *lmd* and *Mef2* mutants compared to the stage matched wild-type embryos. This group contains many genes coding for structural muscle proteins, including *Act57B*, *Act87E* and *betaTub60D* (Figure 1E). As Mef2 expression is also strongly reduced in *lmd* mutant embryos [3], these target genes either depend on input from Mef2 alone or on a combination of Mef2- and Lmd-mediated activation. Other genes are affected differently in the two mutants. Several are upregulated in *lmd* mutants (e.g. *CG9416* and *CG30035*), but are either unchanged or have slightly decreased or increased levels in *Mef2* mutants (cluster II, Figure 1E). In contrast, a third cluster of genes, including *blow*, *goliath (gol)* and *tramtrack (ttk)*, have decreased expression at the late time points in *lmd* mutants and increased expression in *Mef2* mutants, suggesting activation by Lmd and repression by Mef2 (Figure 1E). We note that, although we have used several methods to assess the role of Mef2 in regulating the expression of these genes (see below), we have not found any evidence that Mef2 may act as a transcriptional repressor. Therefore the apparent de-repression of these genes is most likely due to a secondary effect within the *Mef2* mutant embryos. Despite this, the vast majority of genes known to be genetically downstream of Mef2 had significantly reduced expression, indicating that the expression profiling data accurately recapitulates what is expected from genetic studies [11].

### Different combinations of Lmd and Mef2 trigger divergent gene expression

As a complementary approach to assess the regulatory inputs of Lmd and Mef2, we asked if these transcription factors are sufficient, either alone or in combination, to induce target gene expression *in vivo*. The transcription factors were ectopically expressed in parasegmental stripes under the control of the engrailed-Gal4 driver [22] (Figure 2). *Lmd* has been reported to activate *Mef2* expression in the CNS but not in the remainder of the ectoderm under these conditions [16], allowing us to assess the contribution of the two transcription factors independently. As the transcription factors are acting outside of their normal cellular context, this is a stringent assay to investigate regulatory connections.

The transcriptional response of shared target genes to ectopic TF expression was examined using colorimetric *in situ* hybridization (ISH) (Figure 2), and confirmed by double fluorescent ISH (Figure 2, Figure S2). This analysis revealed a range of regulatory responses. We examined three genes that showed reduced expression in both *lmd* and *Mef2* mutant embryos (Figure 1E, cluster I). *betaTub60D* and *Act57B* are ectopically induced by Mef2 alone, but not by Lmd alone (Figure 2A–2B″). As expected, co-expression of both transcription factors also led to ectopic expression (Figure 2A′″, 2B′″). A third gene, *CG5080*, was neither ectopically activated by Lmd nor Mef2 alone (Figure 2C′–2C″). However, when both transcription factors were co-expressed, their combined activity was sufficient to drive ectopic expression, revealing a synergistic regulation of this target gene (Figure 2C′″). Ubiquitous over-expression of *Mef2* using a *daughterless*-Gal4 driver was previously reported to ectopically activate *CG5080* in the head mesoderm [13]. The fact that Mef2 is sufficient to regulate *CG5080* expression in this context, but not in ectodermal stripes, strongly suggests that Mef2 requires additional tissue-specific co-activators also in other tissues of the embryo.

The expression levels of *blow* and *sug* were also strongly reduced in *lmd* mutants, and weakly reduced in *Mef2* mutants (Figure 1E, cluster III). Similar to *CG5080*, neither expression of *lmd* nor *Mef2* alone was sufficient to activate expression of *blow*, *sug* or *sns*, yet ectopic activation was detected upon co-expression of both transcription factors (Figure 2D–2F′″). Although *Mef2* is not required for *sns* expression [23], our data demonstrates that Mef2, in combination with Lmd, is sufficient to activate the expression of *sns* in ectodermal cells. *CG14687* showed the opposite response to *bTub60D* and *act57B*, in that it could be activated by Lmd alone, but not by Mef2 (Figure 2G′–2G″). These data correlate with the expression profiling data, showing a strong requirement of *lmd* activity for *CG14687* expression (Figure 1E, cluster IV). Although *in situ* hybridization is not quantitative, the fluorescent ISH suggests a higher level of expression when both Lmd and Mef2 are co-expressed (Figure 2G, fluorescent panels, Figure S2).

The gene *CG9416* revealed yet another mode of regulatory integration: Mef2 activated ectopic expression of *CG9416* in the absence of Lmd, but this effect appears to be attenuated when both transcription factors were co-expressed (Figure 2H–2H′″, fluorescent panel, Figure S2), indicating opposing regulatory inputs from Mef2 (activation) and Lmd (inhibition). The repressive effect of Lmd is consistent with the dramatic increase in *CG9416* expression in *lmd* mutant embryos (Figure 1E). Finally, the *gol* gene represents the only example tested where even the combination of both Mef2 and Lmd was not sufficient to induce ectopic expression (Figure 2I′″).

In summary, although all genes investigated are directly co-regulated by Lmd and Mef2, ectopically supplying one or both factors revealed considerable flexibility in how information is integrated at each individual locus. In higher eukaryotes, many genes have multiple regulatory elements, which collectively contribute to the complete expression pattern of a gene. To investigate whether the different transcriptional responses to Lmd and Mef2 activity are reflected by the integration of inputs at single enhancers or by the combined activity of multiple *cis*-regulatory elements, we next studied regulatory integration at the CRM level.

### Delimiting enhancer regions co-bound by Lmd and Mef2

Individual enhancer regions in *Drosophila* commonly range from 0.5 to 1 kb in size. The Lmd-bound DNA fragments immunoprecipitated in our ChIP experiments were in a similar size range, however the genomic tiling arrays used in this study limited our resolution to overlapping 3 kb sequences. To achieve higher resolution, we used quantitative real-time PCR to assay the enrichment of shorter sequences within individual 3 kb-bound regions using both the Lmd and Mef2 chromatin immunoprecipitates. In all eight cases examined, the highest enrichment of Mef2 and Lmd binding coincided within a common 0.1 to 1 kb region (data not shown), suggesting that the transcription factors co-occupy a single enhancer element. In addition, each refined sequence was found to contain at least one Mef2 consensus binding site conserved in *Drosophila pseudoobscura* (data not shown).

We tested the ability of the refined Lmd-Mef2-bound regions to regulate expression *in vivo* by generating transgenic reporter lines. All tested enhancer regions specifically activated GFP-reporter expression in the developing muscle (Figure 3 and Figure S1). At stage 11, when both *lmd* and *Mef2* are co-expressed in fusion-competent myoblasts, the enhancers of *blow* (Figure 3A′) and *gol* (Figure 3C′) activated GFP-expression broadly in the visceral and somatic mesoderm. At the same stages, the *CG5080* (Figure 3B′) and *tramtrack* enhancers (*ttk*, Figure 3D′) induced GFP-expression in a subset of myoblasts. At stage 13, when myoblast fusion is in progress, all four enhancers showed almost identical expression patterns throughout the somatic muscle (Figure 3A″–3D″). We also re-examined the spatio-temporal activity of the previously characterized *Act57B* [20], *betaTub60D* [19] and *CG14687* [11] enhancers (Table S2 and Figure S1) and included them in the set of combinatorially-bound enhancers investigated in the remainder of this study.

### Lmd and Mef2 are differentially required for enhancer activity *in vivo*

**Figure 4:**
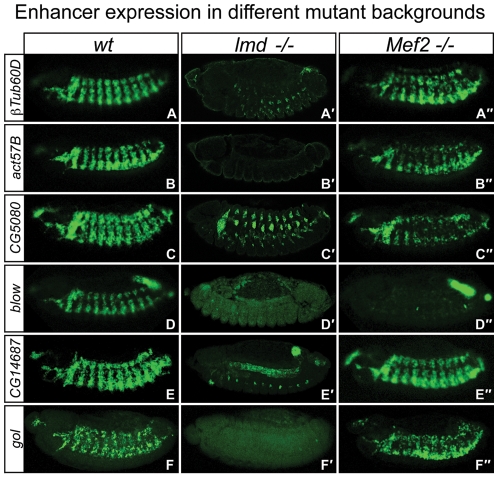
Shared enhancers have differential requirements for Mef2 and Lmd *in vivo*. *In situ* hybridization of GFP-reporter mRNA in (A–F) wt embryos, (A′–F′) homozygous *lmd^1^* mutant embryos and (A″–F″) homozygous *Mef2^P544^* mutant embryos. The intronic *βTub60D* enhancer requires Lmd enhancer activity (A′), but remains active in *Mef2* mutants (A″). Similarly, activity of the *act57B* enhancer is strongly dependent on Lmd (B′), but only mildly reduced in *Mef2* mutants (B″). *CG5080* reporter expression is clearly reduced in both background (C′,C″), while activity of the *blow* enhancer is not detectable in the absence of either Lmd or Mef2 (D′,D″). The *CG14687* enhancer show different requirements in different muscle types: while expression in somatic muscles requires Lmd (E′), expression in the visceral muscle is not affected in neither *Lmd* nor *Mef2* mutants (E″). The *gol* enhancer requires Lmd activity (F′) but is active normally in *Mef2* mutants (F″).

We used the *in vivo* enhancer-reporter lines to study the integration of Lmd and Mef2 regulatory input by comparing CRM activity in wild-type and mutant embryos. Six transgenic reporter lines (Figure 3, Table S2) were placed in the genetic background of *lmd* ^1^ and *Mef2* ^22.21^, two characterized loss-of-function alleles for these transcription factors [2], [3] (Figure 4, Figure S3).

The expression of the *betaTub60D* gene is controlled by several independent *cis*-regulatory modules [19], [24], [25]. An upstream enhancer, 5′ to the *betaTub60D* gene, requires Mef2 activity for its full activation [24]. In contrast, the intronic *betaTub60D* enhancer under study here, although co-occupied by Mef2 and Lmd, appeared unaffected in *Mef2* mutant embryos while having strongly reduced expression in *lmd* mutants (Figure 4A′ and 4A″). The strong reduction in the expression of the *betaTub60D* gene in *Mef2* and *lmd* mutant embryos detected by expression profiling (Figure 1E) therefore reflects the combined activity of at least two enhancers: one strongly responsive to Mef2 levels and a second one depending on Lmd (but not Mef2) for activation.

The *Act57B* enhancer drives GFP-expression in somatic and visceral muscles in wild-type embryos at stage 13 (Figure 4B). This expression was completely lost in *lmd* mutant embryos (Figure 4B′), while *Mef2* mutant embryos showed reduced, but detectable reporter expression, as observed previously [20] (Figure 4B″). In contrast, expression driven by the *CG5080* enhancer was reduced in *lmd* (Figure 4C and 4C′) and to a lesser extent in *Mef2* mutant embryos (Figure 4C and 4C″). Similarly, reporter expression in the somatic muscle driven by the *blow* enhancer was lost in both *lmd* and *Mef2* mutant embryos (Figure 4D–4D″). Enhancer expression in the hindgut visceral muscle persisted in *Mef2* mutant embryos (Figure 4D″), indicating additional tissue-specific input at this enhancer.

The *CG14687* enhancer is activated in somatic and visceral muscle in wild-type embryos (Figure 4E). Expression in somatic muscle required *lmd* expression (Figure 4E′), but is unaffected in *Mef2* mutant embryos (Figure 4E″). Interestingly, expression in the visceral muscle was independent of both *lmd* and *Mef2* expression (Figure 4E′ and 4E″), implicating additional tissue-specific factors in the activation of this enhancer. Both the homeodomain transcription factor *bagpipe* (*bap*) and the fork head domain transcription factor *biniou* are recruited to this enhancer *in vivo* [26] and most likely activate gene expression in this tissue. Finally, the *gol* enhancer required *lmd* activity (Figure 4F and 4F′), but robustly activated gene expression in the absence of *Mef2* (Figure 4F″).

In summary, all six muscle enhancers examined showed reduced activity in one or both mutant conditions, demonstrating that the *in vivo* occupancy of these modules by Mef2 and Lmd has regulatory function. *lmd* mutants generally displayed a stronger reduction in enhancer activity compared to *Mef2* mutant embryos. As Lmd is required to maintain *Mef2* expression, *lmd* mutant embryos are effectively double mutants for both transcription factors. This is reflected by the stronger reduction in enhancer activity in this genetic background and underscores the combinatorial regulation of these enhancers by both transcription factors.

### Cooperative, additive, and repressive effects of Lmd and Mef2 on CRM activity

We next assessed if the combined regulatory inputs of Lmd and Mef2 on these enhancers are integrated in an additive, cooperative or repressive manner. *Drosophila* S2 cells, which express neither endogenous *lmd* nor *Mef2* [27], were used to study the regulatory logic of the different enhancers *in vitro*. Eight regulatory regions that are co-bound by Lmd and Mef2 *in vivo* were placed upstream of a minimal *Hsp70* promoter driving a firefly luciferase reporter and co-expressed with increasing amounts of Lmd and/or Mef2 expression vectors.

**Figure 5:**
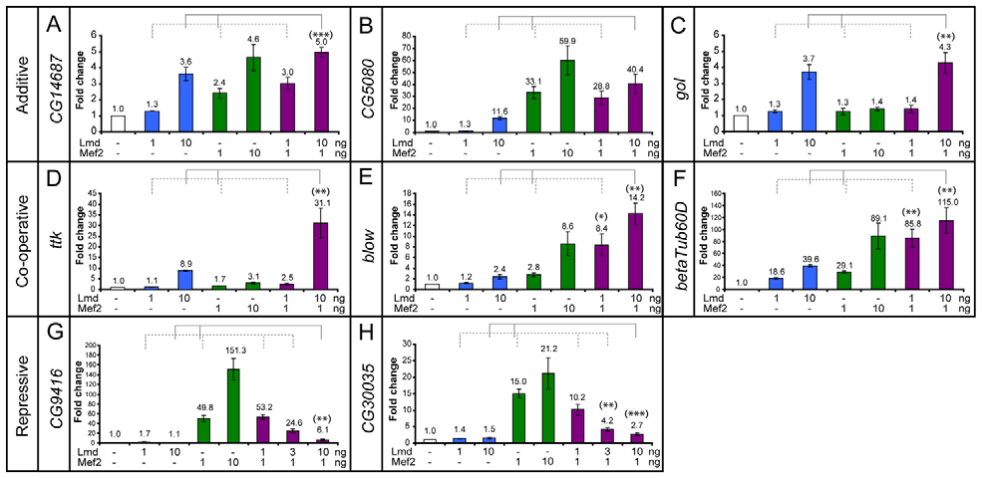
Different modes of co-regulation by Lmd and Mef2 *in vitro*. Minimal regions required for Firefly luciferase reporter activity *in vitro* were identified in the (A) *CG14687*, (B) *CG5080*, (C) *gol*, (D) *ttk*, (E) *blow*, (F) *βTub60D*, (G) *CG9416* and (H) *CG30035* enhancers which were cloned and assayed for activity *in vitro*. Expression plasmids encoding for Lmd or Mef2 proteins were co-transfected with the reporter plasmids and a Renilla normalization control at different concentrations (1 ng/10 ng) alone or in combination (x-axis). Dual-luciferase readout was normalized to reporter-only controls and fold changes are indicated as mean +/− 1 standard error (at least three biological replicates, each done in triplicate). Informative combinations of transcription factor transfections are indicated (brackets). (A–C) Both Lmd and Mef2 can activate the *CG14687*, *CG5080* and *gol* reporters. Co-transfection of both transcription factors leads to roughly additive fold changes (brackets). (D–F) Presence of both Lmd and Mef2 yields higher activity from the *ttk*, *blow* and *Tub60D* enhancers than expected by summing the individual fold changes (brackets), indicating cooperative regulation. (G, H) The *CG9416* and *CG30035* reporters can readily be activated by Mef2, but show reduced activity upon co-transfection with Lmd, revealing its inhibitory influence in this context. The regulatory interactions are highly significant (unpaired, two-tailed student′s t-test, (*) p<0.05, (**) p<0.01, (***) p<0.001).

Co-transfection of either Lmd or Mef2 alone was sufficient to activate the *CG14687*, *CG5080* and *gol* enhancers (Figure 5A–5C), while co-expression of both regulators led to an approximately additive level of reporter activity. For example, transfection of 10 ng of the Lmd expression vector led to a 3.6 fold increase in the luciferase activity driven by the *CG14687* enhancer, while 1 ng of the *Mef2* expression vector led to a 2.4 fold increase in expression. Co-expression of both factors resulted in a 5 fold increase in enhancer activity (Figure 5A).

In contrast, Lmd and Mef2 acted cooperatively to regulate the *ttk*, *blow* and *betaTub60D* enhancers (Figure 5D–5F). For example, expression of either Lmd or Mef2 alone yielded only low levels of reporter gene activity via the *blow* enhancer (Figure 5E). However, co-expression of both transcription factors resulted in much higher levels of activity, indicating a cooperative interaction between Lmd and Mef2 in the context of this enhancer.

Conversely, the *CG9416* enhancer is readily activated by Mef2, but cannot be induced by Lmd (Figure 5G). Instead, co-expression of both transcription factors revealed that Lmd counteracts the positive input of Mef2 to this module, essentially blocking activation by Mef2 in a dose dependent manner. This repressive activity, in combination with the *in vivo* occupancy of Lmd on this enhancer (Figure S1), the increase in *CG9416* gene expression in *lmd* mutant embryos (Figure 1E) and the ability of Lmd to attenuate the ectopic activation of *CG9416* by Mef2 (Figure 2H, fluorescent panels), provides strong evidence that Lmd can provide direct inhibitory input to enhancer activity. Similar to *CG9416*, the expression of endogenous *CG30035* was de-repressed in *lmd* mutant embryos (Figure 1E). The Lmd-Mef2 bound enhancer region close to the *CG30035* locus displayed a similar dose-dependent inhibitory effect of Lmd on Mef2-mediated transcriptional activation (Figure 5H).

Collectively, our results demonstrate that Lmd and Mef2 can induce different regulatory responses depending on the context of the enhancer. This may reflect differences in the relative positioning of Mef2 and Lmd binding to each other or the recruitment of additional unknown factors. As there is no consensus binding site known for Lmd, we used *de novo* motif discovery [28] to identify possible Lmd binding motifs. Since we observed that Lmd and Mef2 are commonly bound within close proximity to each other, we reduced the search space to a 400 bp window around each predicted Mef2 site within the group of 57 co-bound regions. This analysis did not reveal any candidate motifs matching the only known site occupied by Lmd [3], precluding further analysis of individual Lmd binding sites.

## Discussion

Metazoan cells must activate and inactivate the expression of large cohorts of genes in a precise spatio-temporal manner to progress through development. To achieve a molecular understanding of the regulatory networks controlling cellular decision-making, it is essential to understand how inputs from different regulators are being integrated to give rise to defined patterns of gene expression. In this study, we approached this challenge from a genomic perspective by examining the combinatorial input of two key myogenic regulators, Mef2 and Lmd. ChIP-on-chip experiments and expression profiling of loss-of-function mutants were used to systematically identify the direct target genes of the zinc-finger protein Lmd, an important regulator of myogenesis, for which only a single target gene had previously been identified. Integrating these data with data previously obtained for Mef2 revealed that Lmd regulates the majority of its targets in a combinatorial manner together with Mef2. In a few cases these two transcription factors target the same locus through different regulatory regions (e.g. *ladybird-early*, *PAK-kinase* or *short stop*), however in the majority of cases Lmd- and Mef2-binding could be mapped to the same genomic location (Table S1, Table S4). Examining the contribution of both Lmd and Mef2 to regulatory activity, at both the enhancer and gene level, revealed a number of important insights into the contribution of both transcription factors to the myogenic developmental program.

### Combinatorial binding to enhancers leads to diverse regulatory responses

Genes that are co-regulated by the same two (or more) transcription factors are generally expected to have very similar spatio-temporal expression profiles. In fact, this assumption has been used by many studies to computationally predict the location of enhancer elements by searching for common TF binding motifs in the vicinity of clusters of co-expressed genes (or synexpression groups) [29]–[32]. It was therefore surprising when our comparison of experimentally-identified enhancer regions bound by the same two transcription factors uncovered a diverse range of regulatory responses. The 59 genes with enhancer elements co-bound by Lmd and Mef2 at the same stages of development are regulated either in a cooperative, additive or repressive manner depending on the individual enhancers. These data suggest that enhancer regions integrate regulatory inputs more flexibly than previously anticipated. By focusing on individual enhancer elements, we evaluated how Lmd and Mef2 influence regulatory activity in different contexts both *in vivo* and *in vitro*. Combining a number of complementary approaches allowed us to identify three different modes of TF integration at developmental enhancers leading to additive, cooperative or repressive regulation.

### Lmd and Mef2 operate under additive, cooperative, and repressive regulatory logic

Mef2 and Lmd provide an additive positive input to the regulation of the *Act57B* locus. Ectopic Mef2 expression in the ectoderm is sufficient to induce *Act57B* expression, while providing Lmd alone is not (Figure 2B–2B″). Conversely, enhancer-reporter gene expression is completely lost in *lmd* mutant embryos and only slightly reduced in *Mef2* loss-of-function mutant embryos (Figure 4B–4B″). Together, these data reveal a role for both transcription factors at this enhancer. Previous studies demonstrated that the initiation of *Act57B* expression at stage 11 requires Mef2 for its activation. Yet, artificially increasing Mef2 levels at this stage does not lead to premature activation of this locus [13]. Our findings offer an explanation for this result: at this stage of development, combined input from Lmd and Mef2 is required to drive gene expression, while the presence of Mef2 alone is not sufficient to activate transcription. At later stages, when *lmd* expression is lost, Mef2 concentration has increased sufficiently to maintain *Act57B* expression. Conversely, the *CG14687* locus can be activated by ectopic Lmd in the ectoderm, but not by Mef2 alone (Figure 2G′–2G′″) and requires *lmd*, but not *Mef2*, for its expression in the somatic muscle (Figure 4E′–4E″). Combined ectopic expression of the two TFs, on the other hand, leads to a marked increase of reporter signal, again indicating combinatorial positive regulation by both TFs (Figure 2G, fluorescent panels). These findings are supported by the ability of both Lmd and Mef2 to separately activate reporter gene expression *in vitro* and to yield additive reporter activity in combination (Figure 5A).

The *blow* enhancer shows a different mode of regulation and is synergistically activated by both factors. While neither Mef2 nor Lmd alone are sufficient to activate ectopic gene expression *in vivo*, supplying both factors simultaneously leads to robust target gene expression (Figure 2D–2D′″). Assaying for reporter gene activation in the two mutant backgrounds yields a complementary result; Mef2 and Lmd activity is required to activate transcription in the somatic mesoderm via the *blow* enhancer (Figure 4D–4D″). Moreover, the *in vitro* reporter assay reveals a positive interaction between the two proteins (Figure 5E), indicating that the *blow* enhancer functions as a cooperative switch.

Analysis of the *CG9416* enhancer revealed an antagonistic interaction between Lmd and Mef2. While ectopic expression of Mef2 leads to enhancer activation (Figure 2H–2H″), simultaneous expression of Lmd markedly attenuates the transcriptional output from this locus (Figure 2H, fluorescent panels, Figure S2H). This effect can be reproduced *in vitro*: while providing Mef2 alone leads to robust activation of the *CG9416* enhancer, Lmd is not able to activate gene expression (Figure 5G). Instead, Lmd antagonizes the activating input of Mef2 in a concentration-dependent manner. To our knowledge, this is the first example of direct negative regulation by Lmd. To identify additional examples of a repressive role for Lmd, we re-examined the expression profiles of *lmd* and *Mef2* mutant embryos (Figure 1E). *CG9416* is markedly upregulated in *lmd* mutants, but shows reduced expression in embryos lacking *Mef2* (Figure 1E). We selected another direct target gene with similar expression changes in these genetic backgrounds, *CG30035* (Figure 1E) and after determining the limits of the ChIP-enriched region we assayed its ability to drive reporter gene expression *in vitro*. Similar to the *CG9416* enhancer, the *CG30035* enhancer is robustly activated by *Mef2*, and this activation is inhibited by Lmd in a dose-dependent manner (Figure 5H). This provides a second, independent example for Lmd-mediated repression of gene expression.

In summary, starting from a genomic perspective, we have identified a large cohort of genes co-regulated by a pair of tissue-specific transcription factors. Lmd modulates the activity of Mef2 at different enhancers in a context-dependent fashion, allowing for additive, cooperative or antagonistic interactions in the same cells. In this way, the timing and expression levels of Mef2 target genes can be further refined, as exemplified by the *Act57B* locus, which may owe its early activation during embryonic development to the combined activity of both proteins. Lmd shows homology with the Gli superfamily of transcription factors [3], which can act both as transcriptional activators and repressors, depending on proteolytic cleavage regulated by the *hedgehog* signaling pathway. To date, there is no evidence for proteolytic cleavage of Lmd and an irreversible conversion of Lmd from a transcriptional activator to an inhibitor is difficult to reconcile with our observation that Lmd can perform both roles at different loci at the same time, in the same tissue. For the same reason, we also consider it unlikely that Lmd interferes with transcriptional activation simply by binding to Mef2 and sequestering the protein in the cytoplasm. Instead, we propose that Lmd exerts a dominant inhibitory influence over a transcriptional activator, either by locally quenching Mef2's activity or through direct repression of the locus, similar to transcriptional repressors described in other developmental networks [33], [34]. Our results provide a molecular understanding for the genetic observation that restoring Mef2 activity in *lmd* mutant embryos is not sufficient to rescue muscle differentiation [4]. Both transcription factors are required to provide different regulatory inputs to a large number of co-regulated target genes during myogenesis. Their associated enhancers have revealed considerable flexibility in integrating regulatory inputs from these two TFs at individual *cis*-regulatory regions.

## Materials and Methods

### Chromatin immunoprecipitation and DNA amplification

Embryo collections and chromatin immunoprecipitations were performed as described previously [11], [35]. Two antisera were raised against the amino terminus of Lmd and purified from *E. coli* by poly-His tag affinity purification. Four independent staged wild-type embryo populations were collected at 6–8 and 8–10 hrs after egg-laying and fixed with formaldehyde. For each time point, chromatin from all four populations was precipitated with both antisera as well as the respective preimmunesera, leading to a total of 16 reactions (8 mock, 8 anti-Lmd) per time point. DNA amplification, labeling and hybridizations were performed as described previously [11], [35] and dye swaps were included to account for possible dye biases.

### Expression profiling of lmd loss-of-function mutants

The assayed *lmd^1^* [3] line was outcrossed to wild-type flies (Canton S) twice to remove any spurious mutants. Six one-hour embryo collections were assayed in an expression profiling timecourse (between 5 and 11 hours after egg-laying). At each time point, 4 independent populations of *lmd* mutant and stage-matched Canton S embryos were collected and aged. Homozygous mutants were selected with an automated embryo sorter [16], [36]. The staging of all collections was verified by formaldehyde fixation of a small sample to ensure that wild-type and mutant embryos were tightly stage matched. Total RNA was extracted using Trizol (Invitrogen, Carlsbad, US), amplified, reverse-transcribed and labeled as described previously [11].

### Microarray data analysis

For expression profiling analysis, mutant and stage-matched control cDNA was hybridized directly against each other. Raw data was normalized using print-tip LOESS. Differentially expressed genes were identified using Significance analysis of microarrays (SAM) [37]. Genes with a q<1% and a fold change >1.6 (log_2_>0.7 or <−0.7) were considered to be differentially regulated (Table S5). Immunoprecipitated DNA from Lmd-specific or mock precipitations was hybridized against a total genomic reference DNA sample. Sequences significantly enriched by the anti-Lmd-antibodies were identified by comparing rank products [38] and the false-discovery rate was estimated. Only fragments with an FDR <2% and a fold enrichment >1.5 (log_2_ >0.58 or <−0.58) were considered to be significantly enriched (Table S1).

Automatic assignment of ChIP-enriched fragments to target genes was performed as described previously [11]. The majority of regions co-occupied by Mef2 and Lmd was independently assigned to the same target genes using either *Mef2*-mutant or *lmd*-mutant expression profiling data. For a small number of regions, data from this study indicated a more likely target gene than had been assigned previously with Mef2 data alone [11]; in these cases, we chose the updated target prediction for further analysis. A complete list of ChIP-enriched regions, expression profiling results and target assignments are available in Tables S1, S3, S4, S5. All raw microarray data is available from ArrayExpress (Lmd ChIP (E-TABM-895) and *lmd* expression profiling (E-TABM-894). Lmd- and/or Mef2-bound regions and mutant expression data can be visualized at http://furlonglab.embl.de/data/.

### Generation of transgenic reporter strains

Fragments within the following coordinates (based on BDGP genome release 5) were cloned into the pH-stinger (AF242365) vector for germline transformation [39]: chr2R:16831306-16831372 (*actin57B*), chr2R:20197035-20197429 (*betaTub60D*), chr3R:6619371-6620063 (*CG14687*), chr3R:27529661-27530409 (*ttk*), chr2R:3472616-3473387 (*blow*), chr3R:27538572-27539618 (*ttk*), chr2R:8813219-8814579 (*sug*), chr2R:20966587-20969610 (*gol*). For all constructs at least two independent transgenic lines were obtained and assayed. The UAS-*Mef2* line used in this study has been described previously [11]. The UAS-*lmd* line was previously referred to as UAS-*gfl* [16].

### Histological techniques

Double fluorescent *in situ* hybridizations were done as described previously [16]. To minimize experimental differences, the embryo fixations and the *in situ* hybridizations were done in parallel and the confocal imaging was performed with identical laser and gain settings for each gene in the four genetic backgrounds. The following ESTs were used to generate digoxigenin or fluorescein-labeled probes: RE53159 (*betaTub60D*), LD04994 (*act57B*), LD34147 (*CG5080*), LP02193 (*blow*), LD36528 (*sug*), RE74890 (*CG14687*), RE28322 (*CG9416*), GH20973 (*gol*), AT15089 (*twi*) and RE02607 (*wg*). The full-length *sns* cDNA was a kind gift from S. Abmayr. GFP expression in transgenic animals was detected by immunohistochemistry with rabbit α-GFP antibody (Torrey Pines Biolabs) at a concentration of 1:500. Biotinylated secondary antibodies were used in combination with the Vector Elite ABC kit (Vector Laboratories).

### Luciferase reporter assays

*Drosophila* S2 cells were transiently transfected using Cellfectin (Invitrogen). Lmd and Mef2 were expressed from full-length ESTs (LD47926 and GH24154, respectively) in pAc5.1 vector (Invitrogen). The enhancers (coordinates given above) were assayed in a pGL3 luciferase reporter vector (Promega) with an *Hsp70* minimal promoter and the luciferase activity was normalized to Renilla standard (Promega). The total amount of transfected DNA was kept constant by supplementing empty pAc5.1 vector. The measurements were performed according to the supplier's recommendations (Dual-Luciferase Reporter Assay, Promega) with a PerkinElmer 1420 Luminescence Counter.

## Supporting Information

Figure S1 Previously identified enhancer regions are co-occupied by Lmd and Mef2 and reproduce target gene expression *in vivo*. (A–D) Schematic overviews of the tiling array probes covering the (A) *act57B*, (B) *Tub60D*, (C) *CG9416*, and (D) *CG14687* loci. These previously described regulatory regions (black bars) show significant binding of Lmd (blue) and Mef2 (green). (A″–D″) Immuno-histochemistry using an anti-GFP antibody to detect reporter gene expression. All four enriched sequences are able to specifically activate GFP-reporter expression in transgenic embryos in the mesoderm as early as (A′–D′) stage 11, with persistent GFP-presence at (A″–D″) stage 13.
(2.79 MB TIF)

Figure S2 Ectopic expression of Lmd and Mef2 reveals differential regulatory input on target gene expression. Double fluorescent in situ hybridisation (FISH) of the gene of interest (green) and endogenous *wg* gene (red). Ectopic expression (using the engrailed GAL4 driver) should be visible juxtaposed to the *wg* stripe. The *wg* expression was therefore used to ensure that the confocal imaging was performed with identical laser and gain settings for each gene within the four genetic backgrounds. (A–I) FISH of wild-type embryos with probes specific for (A) *βTub60D*, (B) *act57B*, (C) *CG5080*, (D) *blow*, (E) *sug*, (F) *sns*, (G) *CG14687*, (H) *CG9416*, and (I) *gol*, detecting specific expression in the mesoderm. No specific staining was observed in the ectoderm (white brackets). An engrailed-Gal4 driver line was used to ectopically express (A′–I′) UAS-Lmd, (A″–I″) UAS-Mef2-HA or (A″–I″) both UAS-Mef2-HA and UAS-Lmd in ectodermal stripes. Lmd and Mef2 show differential ability to activate ectopic target gene expression (white brackets). Note: The area indicated by the white brackets highlights the ectopic expression.
(9.37 MB TIF)

Figure S3 Enhancer activity in *lmd* and *Mef2* loss-of-function mutant embryos. In situ hybridisations described in Figure 4 were performed by double-staining of GFP (green, first and third column, indicating specific reporter activity) and either endogenous *twist* mRNA (red, in *lmd* mutants) or *lacZ* mRNA expressed from a balancer (red, in *Mef2* mutants) to identify homozygous mutant embryos. Twist expression persists longer in *lmd* mutants (C′. G′, K′, O′, S′, W′) than in wt embryos (A′, E′, I′, M′, Q′, U′). LacZ expression is associated only with heterozygous, balancer containing embryos (B′, F′, J′, N′, R′, T′, X′) (or embryos carrying two balancers).
(4.22 MB TIF)

Table S1 ChIP-on-chip enriched fragments.
(0.08 MB XLS)

Table S2 Overlap of ChIP data with previously characterised enhancer regions.
(0.04 MB PDF)

Table S3 Lmd expression profiling timecourse.
(0.15 MB XLS)

Table S4 Direct targets genes of Lmd and Mef2.
(0.04 MB XLS)

Table S5 Expression profiling of Lmd direct target genes in Lmd and Mef2 mutant embryos.
(0.05 MB XLS)
