# Supplementary figures and images for: Combinatorial Binding Leads to Diverse Regulatory Responses: Lmd Is a Tissue-Specific Modulator of Mef2 Activity
Source: PLoS Genet. 2010 Jul 1;6(7):e1001014. doi: 10.1371/journal.pgen.1001014 (PMC2895655; doi:10.1371/journal.pgen.1001014)

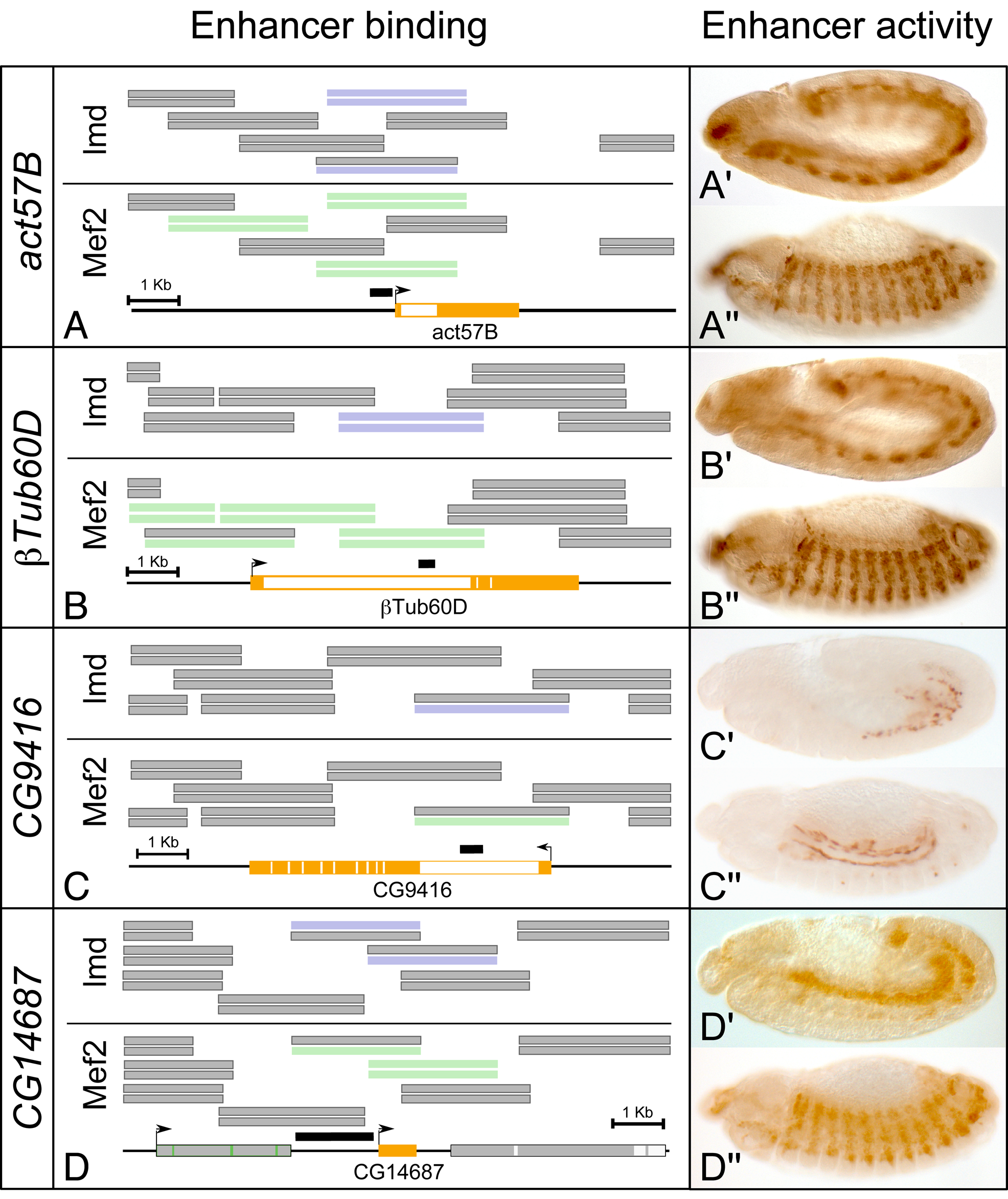

Supplement: Figure S1 — Previously identified enhancer regions are co-occupied by Lmd and Mef2 and reproduce target gene expression in vivo. (A–D) Schematic overviews of the tiling array probes covering the (A) act57B, (B) Tub60D, (C) CG9416, and (D) CG14687 loci. These previously described regulatory regions (black bars) show significant binding of Lmd (blue) and Mef2 (green). (A″–D″) Immuno-histochemistry using an anti-GFP antibody to detect reporter gene expression. All four enriched sequences are able to specifically activate GFP-reporter expression in transgenic embryos in the mesoderm as early as (A′–D′) stage 11, with persistent GFP-presence at (A″–D″) stage 13. (2.79 MB TIF) [file pgen.1001014.s001.tif]

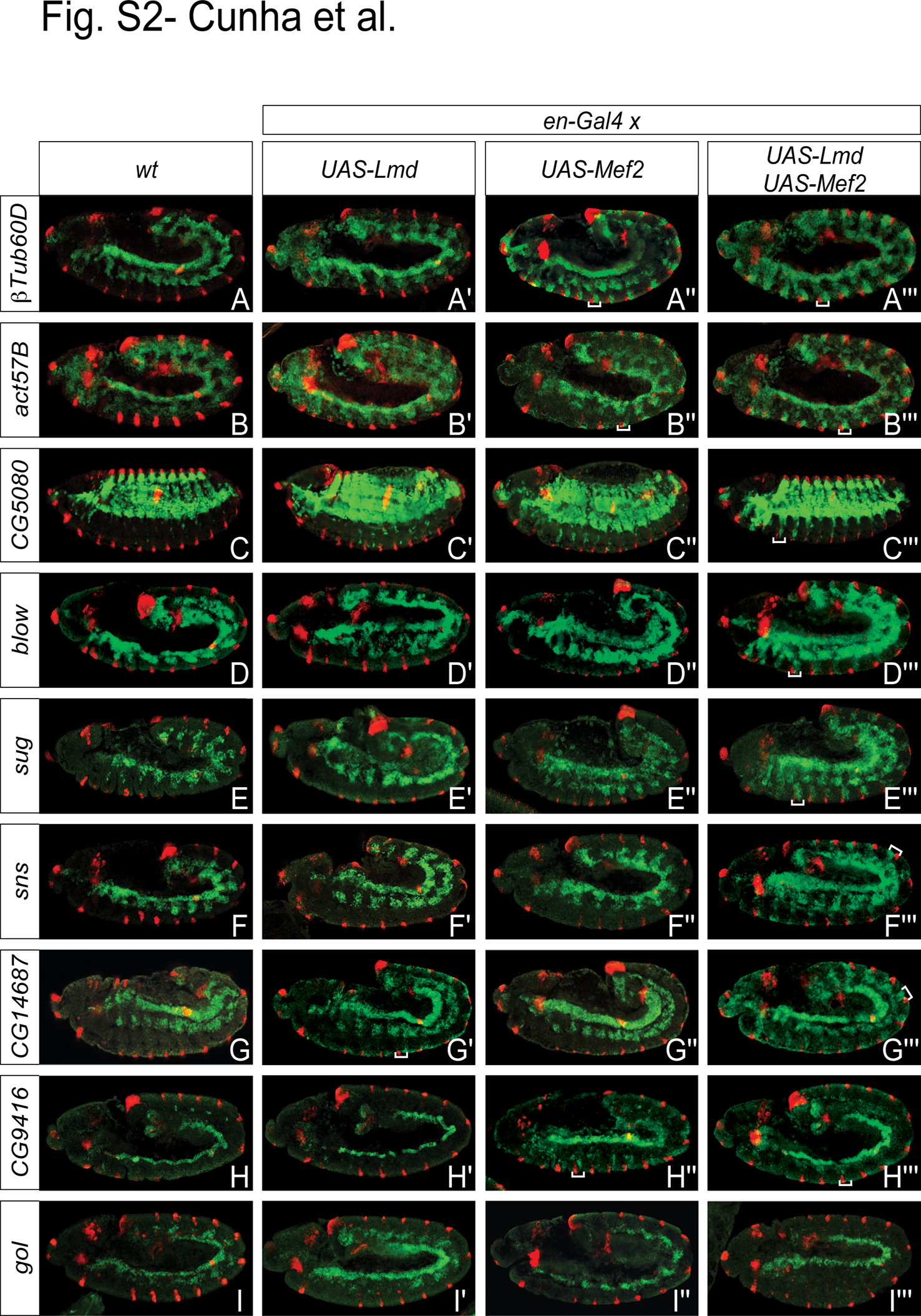

Supplement: Figure S2 — Ectopic expression of Lmd and Mef2 reveals differential regulatory input on target gene expression. Double fluorescent in situ hybridisation (FISH) of the gene of interest (green) and endogenous wg gene (red). Ectopic expression (using the engrailed GAL4 driver) should be visible juxtaposed to the wg stripe. The wg expression was therefore used to ensure that the confocal imaging was performed with identical laser and gain settings for each gene within the four genetic backgrounds. (A–I) FISH of wild-type embryos with probes specific for (A) βTub60D, (B) act57B, (C) CG5080, (D) blow, (E) sug, (F) sns, (G) CG14687, (H) CG9416, and (I) gol, detecting specific expression in the mesoderm. No specific staining was observed in the ectoderm (white brackets). An engrailed-Gal4 driver line was used to ectopically express (A′–I′) UAS-Lmd, (A″–I″) UAS-Mef2-HA or (A″–I″) both UAS-Mef2-HA and UAS-Lmd in ectodermal stripes. Lmd and Mef2 show differential ability to activate ectopic target gene expression (white brackets). Note: The area indicated by the white brackets highlights the ectopic expression. (9.37 MB TIF) [file pgen.1001014.s002.tif]

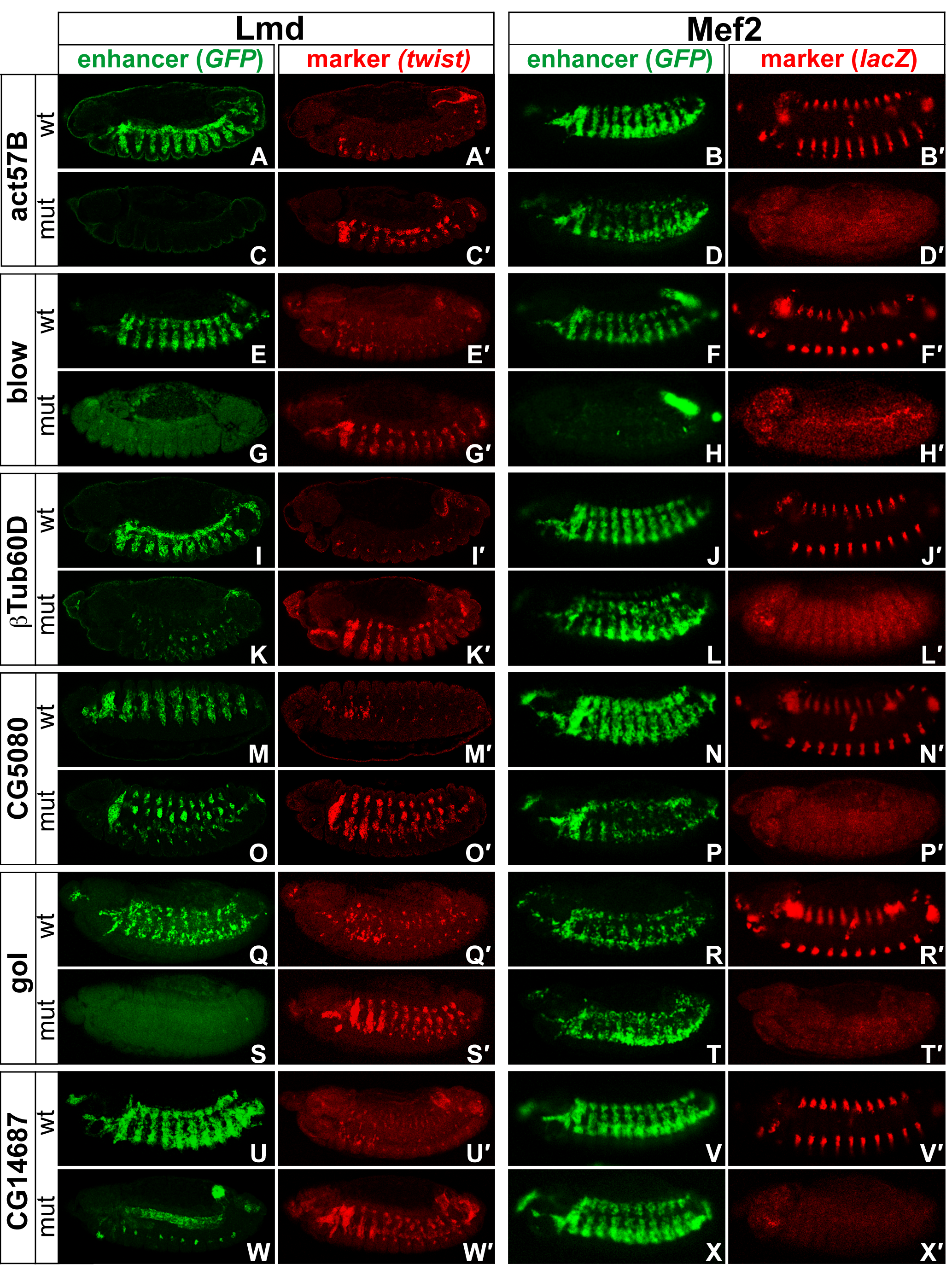

Supplement: Figure S3 — Enhancer activity in lmd and Mef2 loss-of-function mutant embryos. In situ hybridisations described in Figure 4 were performed by double-staining of GFP (green, first and third column, indicating specific reporter activity) and either endogenous twist mRNA (red, in lmd mutants) or lacZ mRNA expressed from a balancer (red, in Mef2 mutants) to identify homozygous mutant embryos. Twist expression persists longer in lmd mutants (C′. G′, K′, O′, S′, W′) than in wt embryos (A′, E′, I′, M′, Q′, U′). LacZ expression is associated only with heterozygous, balancer containing embryos (B′, F′, J′, N′, R′, T′, X′) (or embryos carrying two balancers). (4.22 MB TIF) [file pgen.1001014.s003.tif]
