# Supplementary material for: Combinatorial Binding Leads to Diverse Regulatory Responses: Lmd Is a Tissue-Specific Modulator of Mef2 Activity
Source: PLoS Genet. 2010 Jul 1;6(7):e1001014. doi: 10.1371/journal.pgen.1001014 (PMC2895655; doi:10.1371/journal.pgen.1001014)
Supplement: Table S2 — Overlap of ChIP data with previously characterised enhancer regions. (0.04 MB PDF) [file pgen.1001014.s005.pdf]

Supplemental table S2: Overlap of Lmd-bound regions with enhancers from the RedFly database

(Redfly version 2.0, coordinates from *Drosophila melanogaster* genome ver. 5)

Enriched probe(s)	Chr.	Region start	Region end	Redfly enhancer
D847_2_c5	2L	2169778	2172949	aop_D-lacZ, aop_E-lacZ
D1137_1_a10	2L	12083640	12086257	prd_zebra_enhancer
D600_2_e4, D599_4_h8	2R	5440854	5445049	Mef2_adepithelial_enhancer, Mef2_I-D[L], Mef2_I-D[s], Mef2_I-E
D1055_2_d6, D651_2_b4	2R	16450050	16453029	Act57B_-539/+2
D956_1_a5	2R	19815670	19818422	betaTub60D_beta3-17, betaTub60D_beta3-13, betaTub60D_beta3-14/vm1
D1061_2_c12, D1061_2_d9	3R	21833392	21839172	malpha_PNC
D905_4_d2, D905_2_g8	X	19509152	19515045	dome_dome-MESO
